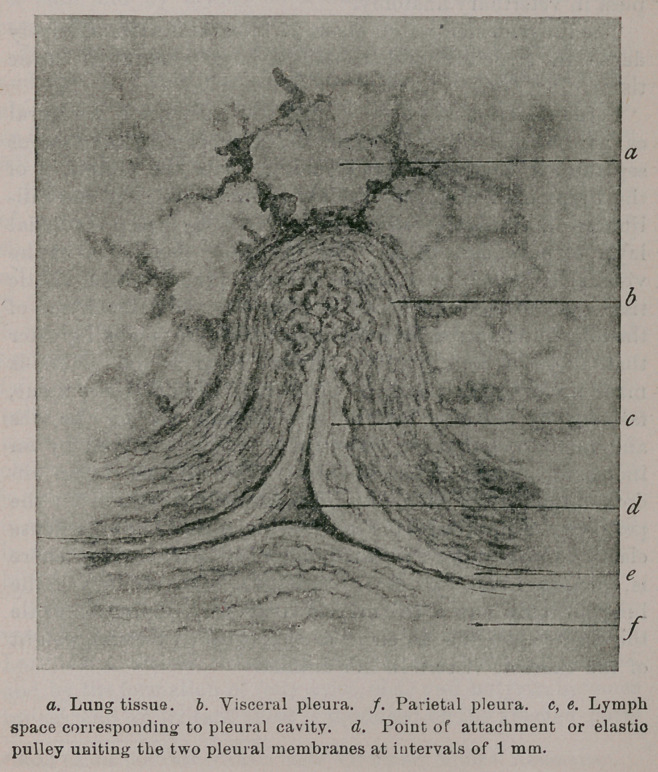# Notes on the Lungs and Digestive Apparatus

**Published:** 1887-04

**Authors:** R. S. Huidekoper


### III.—Notes on the Lungs and Digestive Apparatus.

BY R. S. HUIDEKOPER.

The macroscopic anatomy of the digestive apparatus of the elephant has already been well described. The facial and maxillary muscles descend, and form the lower lip, much as they do in the horse; on the upper jaw they form two fibro, fatty, and irregularly disposed muscular cushions, around the tusks, and transversely between these. From this point the muscles re-arrange themselves into distinct bundles of fibres, to form the trunk, which would answer well to the description given by the older anatomists, of the tongue of the horse, longitudinal, vertical, transverse and circular. The immense nerves and arteries of the trunk lie to the inner and under border of the latteral longitudinal muscles. The stomach is an exaggeration of that of the horse. Length of greater curvature, 1.24 metres; from œsophagus to fundus of cardiac cul de sac, 0 55 m.; lesser curvature, 0.49 m.; circumference, 1 m. The enormous cardiac cul de sac is lined with squamous epithelium, while the stomach, from the œsophageal opening to the pylorus, is lined with columnar cells. The spleen, 1 metre in length and remarkably narrow, hangs by a short epiploon only 12 cm. from the greater curvature of the stomach; the great omentum is folded in all the coils of the gut, forming, when stretched, an immense web some 2 metres in depth. The duodenum passes from the pyloric end of the stomach to the left-hand sub-lumbar region and ends in a small gut about 15 metres in length, which hangs by a mesentery 0.50 m. long. The circumference of the small intestine does not vary much from 37 centimetres. The cæcum is only the length of that of the horse,

*a.* Lung tissue. *b.* Visceral pleura. *f.* Parietal pleura. *c, e.* Lymph space corresponding to pleural cavity. *d.* Point of attachment or elastic pulley uniting the two pleural membranes at intervals of 1 mm.

but has a circumference of 1.10 m., and in it the transverse folds and longitudinal bands are much less marked. The colon is some 9 metres in length, and hangs from a mesentery in spiral coils like that of the small intestine. Microscopic examination of the structure shows the same lining epithelium as in the horse, but the basement membrane contains much more elastic tissue. The termination of the great colon returns to the right-hand side of the end of the duodenum, makes a loop around it to the left, and ends shortly in the rectum. From this disposition, it will be readily seen that the digestive tube is mechanically one of the simplest in veterinary anatomy.

The lungs, which weigh about 27 kilograms, are excessively dense in structure and contain a large amount of elastic tissue.

Former anatomists have denied the existence of a pleural cavity, but this cavity exists, in the form of a series of serous separations covering each alveolus. Over the periphery of the lungs each alveolus presents a small convexity one millimetre in transverse diameter, covered by an endothelial layer of pavement cells. The basement membrane of the visceral endothelium contains a large proportion of elastic tissue, which sends out septa, uniting with the parenchyma of the lungs, and surrounds the alveoli, which are one-half greater than those of the ox. The parietal layer of the pleura is made up of equal quantities of connective and elastic tissue, fastened by its outer face to the internal wall of the ribs and intercostal muscles. The inner face is scolloped by an increased amount of elastic tissue, which is lined by an endothelium, but at each division between the alveoli on the periphery of the lung, this external layer sends down a dense elastic band, to unite with the pulmonary parenchyma. There is, then, an individual serous sack over each alveolus of the lungs, which allows of free movement at every respiration, while the elastic attachments serve to support the excessive weight of the thoracic viscera in this animal.

## Figures and Tables

**Figure f1:**